# A Clinical Practice Guideline on the Timing of Surgical Decompression and Hemodynamic Management of Acute Spinal Cord Injury and the Prevention, Diagnosis, and Management of Intraoperative Spinal Cord Injury: Introduction, Rationale, and Scope

**DOI:** 10.1177/21925682231183969

**Published:** 2024-03-25

**Authors:** Lindsay A. Tetreault, Brian K. Kwon, Nathan Evaniew, Mohammed Ali Alvi, Andrea C. Skelly, Michael G. Fehlings

**Affiliations:** 1 Department of Neurology, NYU Langone Medical Center, New York, NY, USA; 2 Department of Orthopaedics, University of British Columbia, Vancouver, BC, Canada; 3 International Collaboration on Repair Discoveries (ICORD), University of British Columbia, Vancouver, BC, Canada; 4 McCaig Institute for Bone and Joint Health, Department of Surgery, Orthopaedic Surgery, Cumming School of Medicine, 2129 University of Calgary, Calgary, AB, Canada; 5 Institute of Medical Science, University of Toronto, Toronto, ON, Canada; 6 Aggregate Analytics, Inc., Fircrest, WA, USA; 7 Division of Neurosurgery and Spine Program, Department of Surgery, University of Toronto, Toronto, ON, Canada; 8 Division of Neurosurgery, Krembil Neuroscience Centre, Toronto Western Hospital, 7989 University Health Network, Toronto, ON, Canada

**Keywords:** spinal cord injury, decompression, trauma

## Abstract

**Study Design:**

Protocol for the development of clinical practice guidelines following the Grading of Recommendations, Assessment, Development, and Evaluation (GRADE) standards.

**Objectives:**

Acute SCI or intraoperative SCI (ISCI) can have devastating physical and psychological consequences for patients and their families. The treatment of SCI has dramatically evolved over the last century as a result of preclinical and clinical research that has addressed important knowledge gaps, including injury mechanisms, disease pathophysiology, medical management, and the role of surgery. In an acute setting, clinicians are faced with critical decisions on how to optimize neurological recovery in patients with SCI that include the role and timing of surgical decompression and the best strategies for hemodynamic management. The lack of consensus surrounding these treatments has prevented standardization of care across centers and has created uncertainty with respect to how to best manage patients with SCI. ISCI is a feared complication that can occur in the best of hands. Unfortunately, there are no systematic reviews or clinical practice guidelines to assist spine surgeons in the assessment and management of ISCI in adult patients undergoing spinal surgery. Given these limitations, it is the objective of this initiative to develop evidence-based recommendations that will inform the management of both SCI and ISCI. This protocol describes the rationale for developing clinical practice guidelines on (i) the timing of surgical decompression in acute SCI; (ii) the hemodynamic management of acute SCI; and (iii) the prevention, identification, and management of ISCI in patients undergoing surgery for spine-related pathology.

**Methods:**

Systematic reviews were conducted according to PRISMA standards in order to summarize the current body of evidence and inform the guideline development process. The guideline development process followed the approach proposed by the GRADE working group. Separate multidisciplinary, international groups were created to perform the systematic reviews and formulate the guidelines. All potential conflicts of interest were vetted in advance. The sponsors exerted no influence over the editorial process or the development of the guidelines.

**Results:**

This process resulted in both systematic reviews and clinical practice guidelines/care pathways related to the role and timing of surgery in acute SCI; the optimal hemodynamic management of acute SCI; and the prevention, diagnosis and management of ISCI.

**Conclusions:**

The ultimate goal of this clinical practice guideline initiative was to develop evidence-based recommendations for important areas of controversy in SCI and ISCI in hopes of improving neurological outcomes, reducing morbidity, and standardizing care across settings. Throughout this process, critical knowledge gaps and future directions were also defined.

## Introduction

Spinal cord injury (SCI) can be life-altering and result in significant motor, sensory and autonomic dysfunction.^1^ The management of patients with SCI begins at the site of the trauma and requires careful triage by trained emergency responders. The spinal column should be promptly immobilized with the use of a backboard, rigid cervical collar, and supportive blocks in order to reduce motion of the spine and prevent further damage to the spinal cord.^2^ Patients should then be immediately transferred to specialized trauma centers and started on medical therapies that reduce secondary injury and offer neuroprotection.^2^ Hemodynamic management may be important in the early stages of SCI in order to optimize blood flow to tissue surrounding the primary injury that is at risk of infarction.^3^ The injured spinal cord is particularly susceptible to systemic hypotension due to impaired vascular reactivity and loss of auto-regulation. The next step in the management of SCI is assessing whether a patient would benefit from surgical decompression and, if so, how quickly should it be performed.^4^ Finally, individuals with SCI will require intensive rehabilitation for 12 to 18 months in order to promote neurological recovery, optimize functional status, prevent secondary complications, and provide a routine for long-term maintenance of health.^5^ It is imperative to standardize care at every stage of patient management in order to enhance outcomes and improve quality of life. Less commonly, SCI can occur intraoperatively and result from any direct or indirect insult to the spinal cord during induction of anesthesia or patient positioning or as a result of surgical maneuvers or interventions. Intraoperative spinal cord injury (ISCI) is an inherent risk of any spine surgery and can have devastating physical and psychological consequences for patients and their families. Early recognition of intraoperative spinal cord dysfunction through the use of electrophysiologic techniques is essential in order to implement appropriate management strategies that can minimize neurological damage. The management of both SCI and ISCI requires significant health care resources in the form of high-level acute care as well as long-term complication management, and can place substantial financial burden on patients, families, and institutions.^6^ Given the individual and societal impact of SCI, it is critical to establish recommendations in order to standardize care from the scene of the injury through to post-hospital rehabilitation.

Clinical practice guidelines (CPGs) are statements which include recommendations intended to optimize patient care that are informed by a systematic review of the literature and an assessment of the benefits and harms of alternative options.^7,8^ The main objectives of CPGs are to (i) synthesize and translate the highest quality of evidence into practice recommendations; (ii) optimize treatment outcomes and reduce the use of any harmful or unnecessary interventions; (iii) establish standard of care and reduce inappropriate practice variations; (iv) facilitate shared decision making among physicians, patients, and caregivers; and (v) assist policy makers in their decisions about the allocation of health care resources.^9^ In contrast, CPGs are not intended to replace clinical judgement or experience, be the sole source for management decisions, influence reimbursement policies, or be used for performance measures or legal precedents.^8,10,11^ Quality care of patients with SCI and ISCI requires the use of CPGs that combine the highest level of evidence with clinical expertise and patient preferences. It is also imperative that CPGs are periodically reviewed for relevance and updated when new evidence emerges on benefits and harms that may impact recommendations and practice.

The treatment of SCI has drastically evolved over the last century as a result of preclinical and clinical research that have addressed important knowledge gaps, including injury mechanisms, disease pathophysiology, medical management, and the role of surgery. There have been previous CPGs, published by the American Association of Neurological Surgeons (AANS) and AO Spine, that aim to provide recommendations on key components of the management of SCI, including prehospital immobilization, the role of methylprednisolone, cardiopulmonary, and hemodynamic management, the indications for anticoagulation, the utility of magnetic resonance imaging and the timing of surgical decompression.^2,3,12-17^ It is recommended that CPGs are reviewed by the primary sponsor at three years to a maximum of five years following publication. Further criteria for updating a guideline include changes in (i) the evidence related to harm and benefits; (ii) outcomes which would be considered important for decision-making; (iii) ranking of current critical and important outcomes; and (iv) available interventions and resources. Given the availability of more recent research, a multidisciplinary group of clinicians agreed to update the CPGs on timing of surgical decompression and hemodynamic management of SCI. These updated CPGs will incorporate new evidence that is anticipated to be of higher quality in order to better inform current clinical practice.

Currently, there are no systematic reviews or CPGs to assist spine surgeons in the assessment and management of ISCI in adult patients undergoing spinal surgery. Based on the results of a survey of the AO Spine International community, most surgeons performing spine surgeries do not feel comfortable managing ISCI despite considerable training and experience.^18^ Furthermore, 90.6% believed that CPGs for the management of ISCI would be useful and 94.4% reported being either likely or extremely likely to use such CPGs. Given this knowledge gap, there is a pressing need to summarize the existing literature and develop recommendations for the detection and management of ISCI in patients undergoing spinal surgery.

This protocol describes the rationale and proposed methodology for developing CPGs on (i) the timing of surgical decompression; (ii) the hemodynamic management; and (iii) the identification and management of ISCI in patients undergoing surgery for spine-related pathology.

## Rationale and Scope

### Timing of Surgical Decompression

The first section of this guideline aims to define the role and timing of surgical decompression by comparing neurological outcomes and complications between patients treated ultra-early or early vs late. Urgent surgical decompression remains one of the only treatment options for acute SCI and can help restore blood flow to the spinal cord, improve perfusion to tissue at risk, and limit secondary injury. Preclinical evidence has suggested that early surgical intervention can mitigate neural damage caused by persistent compression of the spinal cord and improve neurobehavioral outcomes.^19-21^ Similarly, several clinical studies have indicated that early surgical decompression may enhance neurological recovery in patients with acute SCI.^4^

Across clinical studies, different time thresholds have been used to distinguish between “early” and “late” surgical decompression. The Spinal Trauma Study Group identified the first 24 hours as the most promising time window during which decompression may afford neuroprotection.^22^ In 2017, a CPG was developed by AO Spine to provide evidence-based recommendations for the timing of surgical intervention in patients with SCI, using 24 hours as the cutoff to distinguish early and late decompression.^14^ Based on a systematic review of the literature, a weak recommendation was formulated to suggest early surgery be offered as an option for adult SCI patients regardless of injury level.^14^ For this recommendation, there was controversy among the guideline development group (GDG) about whether the neurological improvement afforded by early surgery was clinically meaningful for patients with SCI. Other limitations to this guideline included (i) insufficient evidence on the differential effectiveness and safety of early vs late surgery in subpopulations; (ii) limited evidence on the cost-effectiveness of early vs late surgery; and (iii) uncertainty surrounding the impact of early vs late surgery on functional outcomes, disability, and quality of life. Furthermore, the level of evidence in the systematic review was low or very low for the majority of findings that informed the CPG, indicating limited confidence in the estimates of effect.

Since the publication of the 2017 CPG by AO Spine, several studies have emerged that assess the impact of early vs late surgical decompression on various outcomes. Previously, the outcomes that were ranked as critical for decision making included improvement in American Spinal Cord Association (ASIA) motor score, ASIA Impairment Score (AIS) by ≥ 2 grades, functional independence measure (FIM), and spinal cord independence measure (SCIM), as well as adverse events. It is anticipated that these scores will also be considered “critical” in the updated version of the guideline and that there will only be minor changes, if any, to the ranking of outcomes. Importantly, the results of recent studies have the potential to change the strength of previous recommendations and therefore impact clinical decisions.

The 2017 CPG by AO Spine defined early surgery as decompression within 24 hours of injury and eliminated studies that evaluated the impact of “ultra-early” surgery on neurological outcomes. In a study by Wilson et al (2015), the mean time from injury to arrival at a definitive care center was 8.1 ± 25.5 hours, while the mean time to surgery was 49.4 ± 65.0 hours. Moreover, 53.3% of patients underwent surgery within 24 hours from injury, while only 34.2% reached the operating room within 12 hours. Given these findings, a recommendation for surgery within 24 hours of injury was deemed to be more feasible than proposing an even narrower time window. Not surprisingly, an increased number of stops at intermediate health centers was one of the most important predictors of delayed surgical intervention. Given that care pathways for individuals with SCI have become more streamlined in certain settings, it may be possible to intervene more expeditiously, especially if there is proof that “ultra-early” surgery is beneficial. It is therefore the endeavor of this guideline to explore different time cut-offs (e.g., <4, <8 or <12 hours) in order to establish the ideal timing of surgical intervention by considering the balance between benefits and harms, cost-effectiveness, and feasibility.

### Hemodynamic Management

The second section of this guideline aims to provide recommendations on the hemodynamic management of acute SCI. A CPG for hemodynamic management is warranted because, aside from surgical decompression, augmenting the mean arterial blood pressure (MAP) is one of the only acute interventions available to clinicians to mitigate further ischemic injury to the spinal cord. Patients with SCI are often managed in an intensive care unit due to increased rates of respiratory insufficiency, cardiac dysfunction and systemic hypotension, and the need for close monitoring.^3^ Systemic hypotension in this patient population may occur from a combination of hypovolemia from concomitant hemorrhage and neurogenic shock. Regardless of the cause, hypotension is likely to be deleterious to the vulnerable spinal cord and to the patient in general.

It has been proposed that reduced MAP can significantly affect the spinal cord by decreasing spinal cord perfusion, altering oxygen delivery to vulnerable tissue, and worsening secondary injury. The injured spinal cord is particularly susceptible to systemic hypotension due to impaired vascular reactivity and loss of auto-regulation. Based on a systematic review of the literature by Ryken et al (2013), volume expansion and targeted elevations in blood pressure were thought to improve neurological outcomes and reduce mortality and morbidity in individuals with SCI.^3^ The findings of this review led to the development of two recommendations on the hemodynamic management of SCI by the AANS/Congress of Neurological Surgeons (CNS): (i) correct hypotension in SCI (systolic BP<90 mmHg) when possible and as soon as possible and (ii) maintain a MAP between 85 and 90 mmHg for the first 7 days following an injury.^3^ There have been challenges in both the interpretation and translation of these guidelines into clinical practice, as well as suggestion that these recommendations may not be appropriate for all patients with SCI.^23,24^ Given this variability in implementation, revising these guidelines is warranted in order to develop recommendations that are more broadly accepted. Furthermore, since 2013, the literature on hemodynamic management of SCI has grown substantially and standards for developing CPGs have evolved.

In 2020, Evaniew et al (2020) conducted a high-quality systematic review that abided by current methodological standards in order to summarize (i) the impact of goal-directed interventions intended to optimize spinal cord perfusion on neurological recovery and adverse events; and (ii) the impact of monitoring techniques, perfusion ranges, pharmacological therapies, and duration of treatment on neurological recovery and adverse events.^25^ This review identified several relevant prospective and retrospective interventional and observational studies that have been published since the development of the 2013 guidelines.^25^ This study also highlighted significant limitations of previous systematic reviews on this topic; specifically, no review assessed the risk of bias of included studies or utilized the GRADE (Grading of Recommendations, Assessment, Development, and Evaluations) approach to evaluate the quality of the overall body of evidence. Furthermore, no other reviews have assessed both the impact of MAP and spinal cord perfusion pressure (SCPP) on neurological outcomes.

According to Evaniew et al (2020), the effect of augmenting MAP on neurological recovery was uncertain.^25^ This conclusion was based on the results of two large cohort studies that demonstrated (i) motor scores did not differ between patient who received vasopressors and those who did not and (ii) drops in MAP were not associated with changes in ASIA motor scores.^26,27^ Other included studies identified MAP support as beneficial but had smaller sample sizes and lower MINORS (Methodological Index for Non-Randomized Studies) scores, indicating higher risk of bias.^25^ Furthermore, low quality of evidence suggested that vasopressor support of MAP is associated with increased rates of arrhythmias, myocardial injuries, and possible posterior reversible encephalopathy syndrome.^25^ These are all factors that must be considered in the approach to hemodynamic management for acute SCI, and justify revisiting the 2013 guidelines that recommended a MAP target of 85–90 mmHg for 7 days. Interestingly, SCPP may result in improved neurological outcomes based on the AIS.^25^ However, evaluating SCPP via intradural catheters represents an invasive monitoring technique with the potential for cerebrospinal fluid (CSF) leak or meningitis. In developing recommendations, it is important to review the strength of evidence supporting the potential benefit of augmenting MAP or SCPP, consider the risks of each approach and evaluate the feasibility and cost-effectiveness of these treatments. A GRADE approach is required to translate this evidence into recommendations that not only incorporate the scientific literature that has become available since the last guideline was published, but also considers multi-disciplinary clinical expertise as well as the perspectives of other key stakeholders. As acute hemodynamic management of SCI is within the purview of many specialties (e.g., anesthesia, intensive care, surgery, rehab medicine), a process that engages these different disciplines is needed to develop truly relevant and applicable guidelines.

### Intraoperative Spinal Cord Injury

The third section of this guideline aims to provide recommendations to assist spine surgeons in anticipating, preventing, diagnosing, and managing ISCI. Although ISCI is uncommon, it is an inherent risk to any spine surgery and can have devastating physical and psychological consequences for patients and their families.^28-30^ Furthermore, an ISCI can impose significant financial costs on a patient, surgeon, as well as an institution and may have severe legal repercussions.^31^ This topic was identified as a key priority for guideline development by an international survey of key stakeholders.^18^ ISCI results from any direct or indirect physiologic insult to the spinal cord, conus medullaris, or cauda equina during surgery, causing temporary or permanent motor, sensory, or autonomic impairment. Proposed mechanisms of ISCI include direct trauma or contusion to the spinal cord, compression of the neural elements by an epidural hematoma or abscess (typically delayed), and spinal cord ischemia secondary to systemic hypotension or from deformity reduction or correction maneuvers.^31^ Furthermore, given the organization of ascending and descending tracts in the spinal cord, postoperative sensory deficits are more likely secondary to direct trauma to the dorsal columns, whereas motor deficits are more likely from spinal cord ischemia and decreased blood flow through the anterior spinal artery.^32,33^

The estimated incidence of ISCI is between 12 and 57 cases per million and varies depending on underlying spinal pathology, regions of involvement, surgical approach, and preoperative neurological deficits.^31,34^ Definitions of ISCI vary across studies and range from quadriplegia to an isolated radiculopathy. Some studies distinguish between nerve root injury and SCI, while others do not and simply report postoperative AIS or Frankel Grades.^35^ There is a pressing need to standardize nomenclature for ISCI in order to better quantify the incidence of major neurological deficits, identify relevant risk factors, implement appropriate preventative strategies, and develop useful and reliable protocols for ISCI management. Furthermore, the variability in the reported incidence of ISCI may be partially attributed to heterogeneity in definitions across studies as well as differences in study design and data collection methods.^36^ Unifying nomenclature and proposing a specific definition for ISCI is an important objective of the guideline development process.

Intraoperative neuromonitoring (IONM) during spine surgery can assess the integrity of the sensory and motor tracts and detect ISCI at a time when action can be taken.^37,38^ IONM has enabled complete resections of spinal cord tumors and more aggressive surgeries for correction of severe and rigid spinal deformities.^37,39^ Somatosensory evoked potentials (SSEPs) evaluate the functional integrity of sensory pathways from the peripheral nerve to the somatosensory cortex via the dorsal column-medial lemniscus; this posterior tract is more likely to be injured from direct trauma.^40^ On the other hand, motor evoked potentials (MEPs) can assess the function of the anterior two thirds of the spinal cord and are useful for detecting damage secondary to spinal cord ischemia.^33,37^ Finally, electromyography (EMG) can be used to provide real time information about selective nerve function.^40^ According to a systematic review by Fehlings et al (2010), the sensitivity of SSEPs, transcranial MEPs (tcMEPs), and EMG for detecting ISCI are 0–100%, 81–100%, and 46%, respectively, while the specificities are 27–100%, 81–100%, and 73%.^38^ Based on this systematic review, there is very low evidence that SSEPs or MEPs in isolation are valid diagnostic tests for detecting ISCI during spinal surgery.^38^ The diagnostic accuracy of unimodal IONM for identifying injury to the motor or sensory pathways, however, may be higher in certain populations such as intradural spinal cord tumors (SSEPs: sensitivity 94.4%, specificity 96.8%; MEPs: sensitivity 95%, specificity 98.9%).^41^ Multimodal IONM (MIONM) has also gained popularity and is now often used in the operating room due to its higher sensitivity (70–100%) and specificity (52.7–100%) for detecting ISCI.^38,42^ While there is consensus that IONM improves outcomes in spinal deformity and oncology cases, there is still controversy with respect to the utility and cost-effectiveness of IONM in other populations such as cervical myelopathy or radiculopathy.^43,44^ The controversy surrounding the decision to use IONM may also stem from the fact that there is only low level of evidence to suggest that IONM reduces the rate of new or worsening neurological deficit.^38^ The results of published systematic reviews should be combined in order to summarize the diagnostic accuracy of IONM and determine when these techniques should be utilized during spine surgery.

Identifying individuals who are at higher risk for ISCI is essential in order to (i) better inform patients during consent discussions, (ii) establish necessary IONM strategies, and (iii) discuss contingency plans with the surgical and anesthesia teams in the event of an IONM alert. Several studies have evaluated risk factors for ISCI in patients undergoing spine surgery for various pathologies. These factors include the presence of preoperative neurological deficits, revision or previous spinal surgery, degree of spinal deformity, surgical technique, number of operated levels, operative duration, estimated blood loss, age, gender, co-morbidities, and body mass index.^29-31,39,45-47^ A systematic review is required to summarize the available evidence on important risk factors for ISCI and determine which populations require closer IONM.

Although IONM allows surgeons to detect potential neurological deficits, it is the response to these alerts that influences outcomes.^38^ IONM can define the nature of the injury and help the surgical team decide whether the surgery can be safely continued.^37,38^ There is variation across studies in terms of IONM warning criteria. Typically, a decrease in the amplitude of SSEPs or MEPs by 50% or an increase in latency by 10% are consistent with spinal cord compromise; however, thresholds of 75% to 80% for amplitude reductions have also been reported.^37,48,49^ Intraoperative checklists have been developed to guide the surgical team’s response to an IONM alert and often include ensuring the IONM system is functioning, confirming the change is not secondary to anesthetic agents, increasing the MAP, decreasing or removing traction weights, administrating methylprednisolone, lidocaine, fluids or warm saline, transfusing blood products and adjusting hardware, screws, and rods.^33,37,48,49^ Unfortunately, these checklists vary across institutions and there is no consensus in terms of how to approach a potential ISCI. Given these knowledge gaps, a further review of the literature and the development of a CPG is needed to outline strategies for responding to an IONM alert.

## Purpose of the Guideline

The main objective of this guideline is to provide evidence-based recommendations on how to best manage patients with SCI and ISCI in order to improve outcomes, minimize permanent injury, and reduce mortality. Specific objectives of this guideline include (i) determine the ultimate timing of surgical decompression; (ii) define specific MAP targets and the duration of MAP augmentation therapy; and (iii) unify the definition of ISCI, define the diagnostic accuracy of IONM techniques, delineate risk factors of ISCI, and propose treatment algorithms for the management of potential ISCI.

## Specific Scope of the Guideline

### Patient Population

This guideline on timing of surgical decompression and hemodynamic management is to be applied in the acute phases of management in adult patients with acute SCI. The guideline on ISCI is to be used in adolescent or adult patients undergoing spine surgery for any spine-related pathology, including infection, trauma, malignancy, deformity, or degenerative disease.

### Definitions

The following definitions are important to understanding the scope of all three guidelines:

• Acute SCI is defined as sudden onset damage or trauma to the spinal cord resulting in loss of tissue integrity, which can lead to impaired motor, sensory, or autonomic function.
• ISCI is defined as a new or worsening neurological deficit attributable to spinal cord dysfunction during the course of spine surgery that is diagnosed intraoperatively by neurophysiologic monitoring or immediately post-operatively based on clinical assessment. Deficits include dysfunction attributable to injury of the spinal cord, conus medullaris, or cauda equina
• Incomplete SCI is defined by the presence of sacral sparing (i.e., sensory and/or motor sparing of the sacral segments S4–5).
• Complete SCI is defined as no sensory or motor sparing in the sacral segments S4–5.
• Central cord syndrome is defined as an incomplete SCI to the cervical central region of the cord, which presents with greater neurological impairment in the upper extremities than the lower extremities.^50^ Central cord syndrome is usually caused by a hyperextension injury in people with previous degenerative pathology. In this guideline, the focus will be on central cord syndrome without instability.
• Conus medullaris is the bundled, tapered end of the spinal cord nerves typically at the level of the first two lumbar vertebra.
• Cauda equina is the collection of nerve roots at the end of the spinal cord. Injury to the cauda equina results in lower motor neuron dysfunction (e.g., hypotonia, hyporeflexia, weakness), sensory loss in lumbar and sacral dermatomes and bladder, bowel, and sexual dysfunction.
• Penetrating injuries to the spinal cord (for some recommendations) are defined as actual penetration of the spinal cord tissue such (e.g., bullet or knife).
• Brown Sequard syndrome is defined as an incomplete SCI characterized by hemiparesis with ipsilateral loss of proprioception and vibration as well as contralateral loss of pinprick and temperature.
• Blunt injury is defined as an insult causing SCI that does not penetrate the cord.
• Early surgery is defined as surgical decompression ≤24 hours after injury, whereas late surgery is defined as surgical decompression >24 hours after injury.
• Ultra-early surgery is defined as surgical decompression in a time frame faster than what is defined as early surgery (i.e., ≤4 hours, ≤8 hours, ≤12 hours)
• AIS is a five grade classification system that assesses spinal cord function.^51^ *Grade A: complete*—no sensory or motor function is preserved in the sacral segments S4–5; *Grade B: sensory incomplete*—sensory but not motor function is preserved below the neurological level and includes the sacral segments, no motor function is preserved more than three levels below the motor level on either side of the body; *Grade C: motor incomplete*—motor function is preserved below the neurological level and more than half of key muscles below the neurological level of injury have a muscle grade less than 3; *Grade D: motor incomplete*—motor function is preserved below the neurological level and at least half of key muscles below the neurological level of injury have a muscle grade greater than or equal to 3; *Grade E: normal*—sensation and motor function are graded as normal in all segments.
• International Standards for Neurological Classification of SCI (ISNCSCI) Motor Score combines the Upper Extremity Motor Score (UEMS) with the LEMS to yield a total score out of 100 (indicates normal).^51^ The function of the following muscles is graded from 0-5: elbow flexors (C5), wrist extensors (C6), elbow extensors (C7), finger flexors (C8), finger abductors (T1), hip flexors (L2), knee extensors (L3), ankle dorsiflexors (L4), long toe extensors (L5), and ankle plantar flexors (S1). A score of 0 = total paralysis, 1 = palpable or visible contraction, 2 = active movement, full range of motion with gravity eliminated, 3 = active movement, full range of motion against gravity; 4 = active movement, full range of motion against gravity and moderate resistance in a muscle specific position, and 5 = active movement, full range of motion against gravity and full resistance in a functional muscle position expected from an otherwise unimpaired person. Both sides of the body are tested: upper extremity right (max = 25), upper extremity left (max = 25), lower extremity right (max = 25), and upper extremity left (max = 25). Upper extremity and lower extremity motor scores are scored independently and are both out of 50.^51^
• ISNCSCI Sensory Score combines Light Touch Scores with Pin Prick Scores.^51^ The sensation of the dermatomes C2-S4/5 is evaluated on both the right and left side of the body. A score of 0 = absent, 1 = altered, and 2 = normal. Both light touch and pin prick are evaluated (light touch right, max = 56; light touch left, max = 56; pin prick right, max = 56; pin prick left, max = 56). Light touch and pinprick scores are scored independently and are both out of 112.
• FIM is an 18-item, clinician-administered scale that evaluates a patient’s independence in eating, grooming, bathing, dressing upper extremity, dressing lower extremity, post-elimination hygiene, bowel management, bladder management, transfers to bed, chair or wheelchair, transfers to tub or shower, transfers to toilet, walking or wheelchair propulsion, stair climbing (all included in FIM Motor Subscore), comprehension, expression, social interaction, problem solving, and memory (all included in FIM soci-cognitive subscale).^52^ The total FIM score ranges from 18 (total dependence) to 126 (total independence); motor scores range from 13 (total dependence) to 91 (total independence); and cognitive scores range from 5 (total dependence) to 35 (total independence).
• SCIM is a 19-item clinician-administered disability assessment tool that evaluates a patient’s ability to perform basic activities of daily living.^53^ The SCIM evaluates three key domains: self-care (6 items related to feeding, bathing, dressing, and grooming), respiration and sphincter management (4 items related to respiration, bladder management, bowel management, use of toilet) and mobility (9 items related to tasks in the room and toilet, indoors, and outdoors). The total score is out of 100, with a lower score indicating greater disability.
• Length of stay is the time from hospital admission to hospital discharge.
• The Minimum Clinically Important Difference is the smallest change in a treatment outcome that a patient or clinician would define as meaningful.^54-56^
• A complication is a treatment-related adverse event.
• MAP is the average arterial pressure throughout one cardiac cycle, systole, and diastole.^57^ During vasopressor therapy, MAP is typically calculated based on the area under the BP waveform, which is recorded invasively by catheter-manometer systems.^58^ Non-invasive estimation of MAP is often performed using automated oscillometric devices (e.g., Dinamap ®) which calculate values based on proprietary algorithms.^58,59^ When evaluating blood pressure using manometry, MAP can be estimated by adding the systolic blood pressure to 2 x the diastolic blood pressure and dividing by 3.^58^
• SCPP is computed by subtracting CSF fluid pressure from MAP.
• Vasopressors are a group of medications used to increase MAP.
• Perioperative is the time period from when a patient is admitted for surgery to when he or she is discharged home or to rehabilitation.
• Sensitivity refers to how often a test is correctly positive in individuals with a particular disease.
• Specificity refers to how often a test is correctly negative in individuals who do not have a particular disease.
• Positive predictive value refers to the percentage of patients with a positive test who actually have the disease.
• Negative predictive value refers to the percentage of patients with a negative test who do not have the disease.
• IONM can be unimodal or multimodal and refers to the electrophysiological test(s) that provide information on the functional integrity of the sensory and motor pathways.
• Unimodal refers to the use of only one electrophysiological test for monitoring during surgery.
• Multimodal refers to the use of more than one electrophysiological test for monitoring during surgery.
• SSEPs are elicited by stimulating a peripheral nerve and are recorded and averaged at a site proximal to the structure at risk, often the spinal cord or the cortex.
• MEPs are evaluated by stimulating impulses at either the spinal cord or motor cortex (tcMEPs) and recording a response at a peripheral nerve or muscle.
• EMG records electrical activity in the peripheral musculature and can be used to detect nerve root injury.
• Spinal cord ischemia is caused by reduced blood supply or increased metabolic demands of the spinal cord.
• Warning criteria are the reductions in amplitude or increases in latency of SSEPs and MEPs that reflect spinal cord compromise.
• Induced hypothermia is the planned reduction of body temperature.

Specific conditions that ***are not covered*** in this guideline include:

• SCI in children or adolescents (i.e., those under 18 years of age).
• ISCI in children less than 11 years of age.
• Chronic SCI, defined as persistence of paralysis for ≥12 months following injury.
• Patients without neurological deficit following trauma.
• Cord compression due to tumor, hematoma, infection, or degenerative disease.
• Inflammatory or infectious myelopathies.
• ISCI secondary to compression by hematoma or abscess in the postoperative period.
• ISCI in patients undergoing surgical procedures distant from the spinal column and cord (e.g., vascular surgery).
• Patients with intraoperative neurological deficits attributed to intracranial pathology (e.g., stroke).

## Aspects of Care Covered by Guideline

The following specific treatments or aspects of care are addressed in this guideline:

• Timing of surgical decompression in patients with acute SCI.
• Hemodynamic management of patients with acute SCI
  • Specify MAP targets and duration of therapy.
• Identification and management of ISCI in patients undergoing spine surgery for any spine-related pathology, including infection, trauma, malignancy, deformity, or degenerative disease.
  • Standardize definitions of ISCI.
  • Define the diagnostic accuracy of IONM for identifying ISCI and outline specific warning criteria in terms of changes in amplitude and latency of SSEPs and MEPs.
  • Identify important risk factors for ISCI.
  • Develop treatment algorithms and evidence-based recommendations on the management of potential ISCI.

Specific treatments or ***aspects of care that are not addressed*** in this guideline include:

• Use of methylprednisolone within 8 hours of injury as per the NASCIS 2 protocol.
• Specific surgical methods for decompression or stabilization of the spine.
• Role of neuroimaging in diagnosing SCI or ISCI, developing management strategies or predicting outcomes.
• Utility of neural prosthetics, cell therapy, or spinal cord stimulators.
• Impact of physical, speech, and respiration therapy on outcomes following SCI or ISCI.
• Direct and indirect costs of SCI or ISCI.
• Long-term neurological, functional, and quality of life outcomes in patients with ISCI.

## Users and Settings

This guideline will facilitate shared decision-making among patients, surgeons, and other healthcare practitioners based on the best available evidence. It is intended to be used by the wide range of clinicians who provide care for patients with SCI or ISCI. Such providers include:

• Pre-hospital providers (e.g., paramedics)
• Emergency department physicians
• In-hospital primary care physicians (e.g., internal medicine, primary care)
• Critical care physicians, including subspecialists in neurocritical and trauma care
• Surgeons with expertise in spinal surgery (e.g., neurosurgeons, orthopedic surgeons, trauma surgeons)
• Spinal cord rehabilitation specialists (i.e., physicians with board certification in SCI Medicine)
• Anesthesiologists
• Other physician groups who may be consulted to care for these patients (e.g., hematologists, vascular surgeons, pain management specialists)
• Allied Health Care providers
• Nurses
• Other stakeholders (e.g., insurers, regulators, legislative decision makers, hospital administration)

This guideline is applicable in the following settings:

• Outpatient Spine Surgery Practice (to facilitate discussions with patients on the risks and benefits of the proposed surgery)
• Site of Trauma and On Route to Trauma Center
• Emergency Department
• Operating Room
• Post-Anesthesia Recovery Unit
• Intensive Care Unit
• Inpatient Wards

## Implementation

Although the evidence-base regarding the effective implementation of CPGs is incomplete, a multipronged approach is generally recommended. Such an approach must include efforts on both a macro (i.e., internationally, nationally, major professional groups) and micro level (i.e., hospital or health care facility, individual health care providers) and must consider the availability of necessary resources (i.e., financial, human, materials) as well as patient values and preferences.

This guideline focuses on the management of SCI and is expected to inform practice based on the best available evidence. It is also expected to facilitate shared decision-making between patients and clinicians, minimize morbidity associated with acute SCI and optimize treatment outcomes. Dissemination of the guidelines will be accomplished at multiple levels:

• Through influential groups and opinion leaders across stakeholder groups (The International Spinal Cord Society, AANS, American Spinal Injury Association, CNS, Academy of SCI Professionals, Canadian Spine Society, Neurocritical Care Society, Wings for Life, Craig Neilsen, Praxis, World Federation of Neurosurgical Societies, North American SCI Consortium, European Association of Neurological Societies, Christopher Reeve Foundation. etc.)
• Guideline review at international meetings
• Publication as a focus issue in the Global Spine Journal
• Advertisement on various stakeholder websites
• AO Spine Core Competency/Curriculum
• Webinars to target broader audiences (consider taping any formal presentations made at professional meetings)
• Submission to Emergency Care Research Institute (https://www.ecri.org/) for dissemination of these guidelines.

## Summary of Contents

Three systematic reviews, two scoping reviews and one narrative review were conducted to summarize the current body of evidence and inform the guideline development process. Table 1 highlights the key questions addressed in each review and synthesizes the main results. The following are the recommendations developed during the guideline process:

**Table 1.** Evidence Summary from the Systematic Reviews used to Develop our Recommendations.

Title	Key Clinical Questions	Main Results
Timing of Decompressive Surgery in Patients with Acute Spinal Cord Injury: Systematic Review Update	**Key Question 1**: What is the effectiveness of early decompression (≤ 24 hours) compared with late decompression (>24 hours) or conservative therapy based on clinically important changes in neurological status? What is the effectiveness of ultra-early decompression compared with other “early” time frames up to 24 hours (e.g., < 8 hours vs ≥ 8 hours but <24 hours)? **Key Question 2:** How does timing of decompression influence other functional outcomes or administrative outcomes? **Key Question 3:** What is the safety profile of early decompression compared with late decompression? **Key Question 4**: Does early decompression have differential efficacy or safety in specific subgroups of patients? **Key Question 5**: What is the cost-effectiveness of early decompression compared with late decompression?	*Very Low:* Early surgery was associated with improved total ASIA motor score in the short-term (≤ 6 months) compared with late surgery. *Moderate*: Early surgery was associated with improvement in total ASIA motor score at 12-months compared with late surgery. *Moderate:* Early surgery resulted in a two-fold or greater likelihood of achieving ≥ 2 grade improvement on AIS at 6- and 12-months compared to late surgery. *Very Low:* Firm conclusions on the effectiveness of ultra-early surgery (<4 or <8 hours) to improve AIS by ≥ 2 grades by 6 months were not possible. *Very Low:* Firm conclusions on the effectiveness of ultra-early surgery (<5 or <12 hours) to improve AIS by ≥ 2 grade by 12 months were not possible. *Very Low*: Firm conclusions on the effectiveness of early surgery compared with conservative treatment in patients with pre-existing cervical spinal stenosis who experienced incomplete traumatic SCI were not possible. *Very Low*: Although early surgery tended to improve FIM motor scores compared with late surgery, the estimates should be viewed cautiously given quality of studies and lack of precision. *Low*: Pooled estimates suggested that patients who undergo early surgery (≤24 hours) have a shorter hospital length of stay (∼3 days) compared to late surgery (>24 hours). The impact of this difference on costs or other outcomes is unknown. *Very Low*: There was insufficient evidence to determine the impact of timing of surgery on length of stay in rehabilitation. *Very Low:* There was insufficient evidence to determine whether hospital length of stay differs between ultra-early and early surgery groups. *Moderate*: There was no difference in the rate of major complications between patients undergoing early compared to late surgery. *Low*: There was no difference in rate of mortality between early and late surgery. *Low*: There was no difference in rate of surgical-device related complications between early and late surgery. *Low:* There was no difference in the rate of sepsis between early and late surgery. *Low:* There were fewer decubitus ulcers in patients undergoing early surgery compared to late; however, results were within the limits of chance. *Very Low*: There was no difference in rate of neurological deterioration between early and late surgery. *Low:* There were fewer cardiopulmonary complications in patients undergoing early surgery compared to late; however, results were within the limits of chance. *Low*: There were fewer tracheostomies required in patients undergoing early surgery compared to late; however, results were within the limits of chance. *Very Low:* There was no difference in the rate of mortality between ultra-early and early surgery for all studied thresholds (4- and 8 hours). *Very Low:* There was no difference in the rate of CSF leak between ultra-early (<8 hours) and early (>8 hours, <24 hours) surgery. *Very Low*: There was no difference in rate of neurological deterioration between ultra-early (<12 hours) and early (>12 hours, <24 hours) surgery.
Interventions to Optimize Spinal Cord Perfusion in Patients with Acute Traumatic Spinal Cord Injuries: A Systematic Review Update	**Key Question 1:** In patients with acute traumatic SCI, what are the effects of goal-directed interventions to optimize spinal cord perfusion on extent of neurological recovery and rates of adverse events at any time point of follow-up? **Key Question 2:** In patients with acute traumatic SCI, what are the effects of particular monitoring techniques, perfusion ranges, pharmacological agents, and durations of treatment on extent of neurological recovery and rates of adverse events at any time point of follow-up?	*Very Low:* The effect of MAP support on neurological recovery is uncertain. The majority of evidence favoring MAP support was derived from uncontrolled studies. Two of the largest studies failed to identify consistent benefit of MAP augmentation on neurological recovery. *Very Low:* The use vasopressors for MAP support may be associated with increased rates of cardiac arrhythmias, myocardial injury, acidosis, skin necrosis, and other adverse events. Overall, rates of cardiac adverse events range from 40% with phenylephrine to 76% with dopamine. The most common types of cardiac complications were tachycardias, atrial fibrillation, troponin elevations, and bradycardias. One of the largest studies failed to identify an association between vasopressor use and adverse events. *Very Low:* Increased SCPP may be associated with improved neurological recovery. Increased SCPP resulted in neurological recovery by at least one AIS grade at up to 6 months in the largest case-controlled study^38,39^ and up to 12 months in two uncontrolled studies. However, a third study failed to confirm an association between SCPP and neurological recovery at a mean follow-up of 17 months. *Low*: CSF drainage via lumbar intrathecal catheters was not associated with improved neurological recovery in one small RCT. *Very Low:* SCPP monitoring via intradural pressure probes at the anatomical site of injury was associated with CSF leakage requiring revision wound closure (7%) and asymptomatic pseudomeningocele (19%–54%).^26,33^ *Low:* SCPP monitoring and/or CSF drainage via lumbar intrathecal catheters was not associated with any adverse events. *Very Low:* No studies directly compared the effects of implementing different MAP targets on neurological outcomes. Fourteen studies reported on associations between neurological recovery and augmenting MAP to 70-95 mmHg. *Very Low:* No studies directly compared the effects of implementing different SCPP targets on neurological outcomes. In a large prospective observational study, episodes of SCPP below 50 mmHg were associated with failure to improve by at least one AIS grade at 6 months (OR: .9, 95% CI 0.8 to 1.0, P = .03). In a second study, maintaining a SCPP of 60-65 mmHg was associated with improved neurological recovery. A third study on 15 patients reported improvements on AIS when SCPP was ≥65 mmHg.^60^ A final study on 19 patients reported a reduction in motor scores when SCPP was >110mmHg. *Very Low:* No studies directly compared the effects of monitoring SCPP via lumbar intrathecal catheters vs intraspinal pressure probes at the level of the injury on neurological outcomes. *Very Low:* Three studies compared either adverse events or neurological recovery among vasopressors used to support MAP. Dopamine was associated with higher rates of adverse events compared to norepinephrine or other agents. A single study reported no difference in neurological improvement between patients who received dopamine vs phenylephrine. *Very Low:* No studies directly compared the effects of specific durations of MAP or SCPP support on neurological outcomes. Fourteen studies reported on the association between neurological recovery and targeting MAP or SCPP goals for 3–7 days post-injury.
Definition, Frequency and Risk Factors for Intra-Operative Spinal Cord Injury: A Knowledge Synthesis	**Key Question 1:** What are the risk factors for the development of an ISCI?	*Moderate:* Increasing age was associated with increased odds of experiencing ISCI in patients undergoing deformity surgery. *Low:* It is unclear whether age is a risk factor for ISCI in patients undergoing spine surgery for mixed pathology. *Very Low: A*ge was not a risk factor for ISCI in patients undergoing surgery for degenerative spine disease. *Low:* Males had increased odds of experiencing an ISCI in patients undergoing spine surgery for mixed pathology. *Very Low:* Sex was not a risk factor for ISCI in patients undergoing surgery for degenerative spine disease. *Low:* It is unclear whether hypertension is a risk factor for ISCI in patients undergoing spine surgery for mixed pathology. *Low*: Diabetes was not a risk factor for ISCI in patients undergoing spine surgery for mixed pathology. *Low to Very Low:* BMI or obesity were not risk factors for ISCI in patients undergoing spine surgery for mixed pathology (low) or degenerative spine disease (very low). *Low:* Depression was not a risk factor for ISCI in patients undergoing spine surgery for mixed pathology. *Very Low:* Charlson Comorbidity Index was not a risk factor for ISCI in patients undergoing surgery for degenerative spine disease. *Low:* Dyslipidemia was not a risk factor for ISCI in patients undergoing spine surgery for mixed pathology. *Very Low:* Abnormal pulmonary function was associated with increased odds of experiencing an ISCI in patient undergoing deformity surgery. *Very Low*: Preoperative neurological status was not a risk factor for ISCI in patients undergoing deformity surgery. *Moderate:* Better preoperative AIS was associated with decreased odds of experiencing an ISCI in patients undergoing spine surgery for mixed pathology. *Very Low*: Presence of compressive myelopathy prior to surgery was associated with increased odds of experiencing an ISCI in patients undergoing surgery for degenerative spine disease. *Moderate:* It is unclear whether estimated blood loss is a risk factor for ISCI in patients undergoing deformity surgery. *Very Low*: Estimated blood loss was not a risk factor for ISCI in patients undergoing surgery for degenerative spine disease. *Moderate:* Number of spinal levels involved was not a risk factor for ISCI in patients undergoing deformity surgery. *Very Low*: Number of levels fused was not a risk factor for ISCI in patients undergoing surgery for degenerative spine disease. *Low:* An increasing number of involved segments was associated with increase odds of experiencing an ISCI in patients undergoing spine surgery for mixed pathology. *Low:* Lumbar-level osteotomy was associated with increased odds of experiencing an ISCI in patients undergoing deformity surgery. *Very Low:* Ponte-osteotomy was associated with increased odds of an IONM alert in patients undergoing deformity surgery. *Low:* 3-column osteotomy was not a risk factor for ISCI in patients undergoing deformity surgery. *Very Low*: Operation type (emergency vs elective) was not a risk factor for ISCI in patients undergoing surgery for degenerative spine disease. *Very Low:* Operation time was not a risk factor for ISCI in patients undergoing surgery for degenerative spine disease. *Very Low:* Use of IONM was associated with decreased odds of experiencing an ISCI in patients undergoing surgery for degenerative spine disease. *Moderate:* Increased coronal DAR was associated with increased odds of experiencing an ISCI in patients undergoing deformity surgery. *Very Low*: Increased curve magnitude was associated with increased odds of experiencing an ISCI in patients undergoing deformity surgery.
Role of Neuromonitoring for Detecting Intraoperative Spinal Cord Injury During Spinal Surgery: A Systematic Review and Meta-analysis	**Key Question 1**: What is the accuracy of neurophysiological monitoring for diagnosis of ISCI compared with immediate postoperative clinical assessment?	*Low*: A total of 52 studies with 18,076 patients presented data for SSEP. Overall, the sensitivity of SSEP was 67.5% (95% CI 50.9–80.6%), while the specificity was 96.8% (95% CI 94.8–98.1%). *Low*: A total of 75 studies with 79,545 patients presented data for MEP. Overall, the sensitivity of MEP was 90% (95% CI 86.1–92.9%), while the specificity was 95.6% (95% CI 94–96.7%). *Low*: A total of 16 studies with 7004 patients presented data for EMG. Overall, the pooled sensitivity for EMG was 48.3% (95% CI 31.4–65.6%), while the pooled specificity was 92.9% (95% CI 84.4–96.9%). *Low*: A total of 69 studies with 58,325 patients presented data for MIONM. Overall, the sensitivity of MIONM was 91% (95% CI 86–94.3%), while the pooled specificity was 93.8% (95% CI 90.6-95.9%).

### Timing of Surgery

**Population:** Adult patients with acute SCI.

**Key Question 1:** Should we recommend early decompressive surgery (≤24 hours after injury) for adult patients with acute SCI regardless of injury severity and neurological level?

**Recommendation 1***:* We recommend that early surgery be offered as an option for adult patients with acute SCI regardless of level.

**Quality of Evidence:** Moderate

**Strength of Recommendation:** Strong

**Population:** Adult patients with acute SCI.

**Key Question 2:** Should we recommend ultra-early decompressive surgery for adult patients with acute SCI regardless of injury severity and neurological level?

**Statement:** A recommendation for “ultra-early” surgery could not be made on the basis of the current evidence because of the small sample sizes, variable definitions of what constituted “ultra-early” and the inconsistency of the evidence.

### Hemodynamic Management

**Population**: Adult patients with acute SCI.

**Key question 1**: Should we recommend the augmentation of MAP to at least 75–80mmHg and not higher than 90–95mmHg in order to optimize spinal cord perfusion in acute traumatic SCI?

**Recommendation 1**: We suggest the augmentation of MAP to at least 75–80mmHg but not higher than 90–95mmHg in order to optimize spinal cord perfusion in acute traumatic SCI.

**Quality of Evidence**: Very Low

**Strength of Recommendation**: Weak

**Population**: Adult patients with acute SCI.

**Key question 2**: Should we recommend the augmentation of MAP for a duration of 3–7 days in order to optimize spinal cord perfusion in acute SCI?

**Recommendation 2**: We suggest the augmentation of MAP for a duration of 3–7 days in order to optimize spinal cord perfusion in acute SCI.

**Quality of Evidence**: Very Low

**Strength of Recommendation**: Weak

**Population**: Adult patients with acute SCI.

**Key question 3**: Should we recommend the use of a specific vasopressor in order to achieve MAP-directed goals in patients with acute SCI?

**Statement:** The decision should be left to the attending physician in terms of what vasopressor or inotrope to use in order to achieve MAP-directed goals in patients with acute SCI.

### Intraoperative Spinal Cord Injury

**Population**: Adult patients undergoing spine surgery.

**Key Question 1:** Should we recommend that intraoperative neurophysiologic monitoring be employed for high risk patients undergoing spine surgery?

**Recommendation 1:** We recommend that intraoperative neurophysiologic monitoring be employed for high risk patients undergoing spine surgery.

**Quality of Evidence**: Low

**Strength of Recommendation:** Strong

**Population:** Adult patients undergoing spine surgery.

**Key Question:** Should we recommend that patients at “high risk” for ISCI during spine surgery be proactively identified, that after identification of such patients, multi-disciplinary team discussions be undertaken to manage patients, and that an intraoperative protocol including the use of IONM be implemented?

**Recommendation 2:** We suggest that patients at “high risk” for ISCI during spine surgery be proactively identified, that after identification of such patients, multi-disciplinary team discussions be undertaken to manage patients, and that an intraoperative protocol including the use of IONM be implemented

**Quality of Evidence:** Very Low

**Strength of Recommendation:** Weak
